# When to Fill Teeth

**Published:** 1897-01

**Authors:** I. C. Edington

**Affiliations:** Rochester. N. Y.


ARTICLE VI.

### WHEN TO FILL TEETH.

BY I. C. EDINGTON, D. D. S., ROCHESTER. N. Y.

Read before the Seventh Distr ct Dental Society, April, 1896.

In speaking of this subject a few days since, I was made aware of being dangerously near the Line, and am so unfortunate as not to have heard him read that paper on "The Preparation of Teeth—not cavities." So if any one's toes are trod on, or their ground harrowed, it is unintentional. We wish to call special attention to that form of caries known as white decay, or rapid decay,—which we so often find and fail to stop,—and the time for filling cavities thus disposed. We do not presume to take up the etiology of white decay or its cure; but to give some practical thought as to its treatment, and the time for filling teeth thus affected. This class of teeth are by no means uncommon in this age of prepared foods and lively struggle for existence, and in which very many of our population do not take time to properly masticate their food or cleanse their teeth. We might call attention in passing, that very many of our foods do not need mastication, this act being accomplished in their preparation.

A word about what may cause this too prevalent evil white decay, and the habits of people who present their condition: I find this condition usually in mouths subjected to uncleanliness, persons who are in the habit of lunching between meals, and cleansing their teeth in the morning before breakfast, or when they "dress up," but the white decay is rare where the teeth are thoroughly cleansed just before retiring. The soft foods—or foods classed as hydrocarbonates—when allowed to remain on and between the teeth, especially at night, are in my opinion responsible for much of this white decay. Such foods form the hotbed of decay and the base of supply for lactic acid.

In the past year I have questioned patients presenting this condition, and invariably find that they do not cleanse their teeth at night just prior to retiring; that they are in the habit of lunching or are habitual cousumers of candy, or make a practice of taking a glass of milk on retiring. The girls at the typewriter; the maids in the kitchen; the mother with a number of children; the lean, slim person; the children who are continually munching on ginger-bread with a cookie in hand, represent the class in which the greatest per cent. of this white decay is to be found. Where there is a general tendency to the conditions named, I think it improper to attempt filling of a permanent character. If the condition is allowed to remain, decay will be sure to follow in the near future and loss of fillings ensue. What the public need is a better education regarding the hygiene of the mouth. Probably many persons brush their teeth enough, but at the wrong time. If they would confine their energies in that direction, to thorough cleansing just before retiring at night, much tooth structure would be saved.

Cleansing the teeth at night is one of the salient points I wish to make. At night when the muscles are at rest and nature's tooth-wash, the saliva, does not flow, with a little food on and between the teeth, together with moisture and warmth, we have as fine a culture medium for the decay-producing micro-organisms as the most fastidious bacillus could wish.

When are we to fill such teeth? Certainly not until we have changed their surroundings. One of the most important duties of a dentist is to impress upon the minds of his patients the importance of their co-operation with him. He should instruct them how to properly cleanse their teeth and suggest a wash or powder, as the case may indicate. Very frequently we find in the mouth more or less acid. In that case milk of magnesia is the thing to use. It should be used to rinse the mouth after the teeth have been cleansed as thoroughly as possible. A desert spoonful should be rinsed thoroughly around the teeth and gums and allowed to remain. The milk of magnesia should be used because of its sustained alkalinity. I know of no other preparation that is equal to it. It is cheap, pleasant to use and efficacious. I have in mind a case which will illustrate my point as well as any I can think of. A lady, twenty-four years old, housekeeper for her father. She applied to have her teeth filled, and they needed it. Almost every tooth was effected. They did not look it at a glance, but on close examination showed caries of a soft nature, in color, white, and very rapid in action. A line of white cheesy decay extended between the enamel and dentine to a depth surprising. The dentine was soft and very sensitive. Patient said she cleansed her teeth every day after dinner when she dressed for the afternoon, sometimes in the morning, but never on retiring for the night. Her teeth were extremely sensitive to thermal changes, also to sweets and salt. I removed the soft decay from some of the worst cavities, wiped with carbolic acid and filled with temporary stopping, directing that the mouth be rinsed with milk of magnesia three times a day, after breakfast, dinner, and upon retiring, subsequent to a thorough cleansing of the teeth, and to report in one week.

At the second visit the condition was much improved. The white decay had taken on a light brown color. The patient remarked she was not aware that she had so many teeth needing to be filled. By changing the color the cavities were made visible. Treatment continued and at the end of four weeks I filled with soft filling, and shall fill with gold in about a year.

The teeth in this case were below the medium in structure, well formed and regular in the arch, and I believe with proper care they are destined to remain and do service for many years, I think in this case gold could not have been used and made to serve the purpose. And I do think that to save this class of teeth, the condition must be changd and the habit of properly cleansing the teeth be formed before the permanent filling is attempted. We could mention numerous cases of a similar character, but one will suffice.

To conclude : Do not attempt to fill teeth suffering with white decay until you are convinced that their surroundings are changed or will be. Explain to patients the necessity of properly cleansing their teeth and the benefits to be derived therefrom. Get them to promise to spend at least three minutes a day in cleansing their teeth, and if they cannot cleanse them but once a day, let that once be just before retiring for the night, thereby having the mouth clean for the greater number of hours of the twenty-four. Under these circumstances, we may expect more of our fillings and our work in general, to be a credit to us and a joy to our patients.—*Odontographic Journal.*

---

